# Pain, Parental Involvement, and Oxytocin in the Neonatal Intensive Care Unit

**DOI:** 10.3389/fpsyg.2019.00715

**Published:** 2019-04-02

**Authors:** Manuela Filippa, Pierrick Poisbeau, Jérôme Mairesse, Maria Grazia Monaci, Olivier Baud, Petra Hüppi, Didier Grandjean, Pierre Kuhn

**Affiliations:** ^1^Division of Development and Growth, Department of Paediatrics, Gynaecology and Obstetrics, University of Geneva, Geneva, Switzerland; ^2^Neuroscience of Emotion and Affective Dynamics Laboratory, Department of Psychology and Educational Sciences, Swiss Center for Affective Sciences, University of Geneva, Geneva, Switzerland; ^3^Department of Social Sciences, University of Valle d’Aosta, Aosta, Italy; ^4^Centre National de la Recherche Scientifique, Institute for Cellular and Integrative Neurosciences, University of Strasbourg, Strasbourg, France; ^5^INSERM U1141 Protect, Paris-Diderot University, Paris, France; ^6^Division of Neonatology and Paediatric Intensive Care, Department of Paediatrics, Gynaecology and Obstetrics, Universtiy of Geneva, Geneva, Switzerland; ^7^Service de Médecine et Réanimation Néonatale, Hôpital de Hautepierre, Centre Hospitalier Universitaire de Strasbourg, Strasbourg, France

**Keywords:** prematurity, pain, parents, early separation, early contact

## Abstract

Preterm infants (PTI) typically experience many painful and stressful procedures or events during their first weeks of life in a neonatal intensive care unit, and these can profoundly impact subsequent brain development and function. Several protective interventions during this sensitive period stimulate the oxytocin system, reduce pain and stress, and improve brain development. This review provides an overview of the environmental risk factors experienced by PTI during hospitalization, with a focus on the effects of pain, and early maternal separation. We also describe the long-term adverse effects of the simultaneous experiences of pain and maternal separation, and the potential beneficial effects of maternal vocalizations, parental contact, and several related processes, which appear to be mediated by the oxytocin system.

## Introduction

Recent improvements in neonatal intensive care units (NICUs) have contributed to the increased survival rates of extremely PTI (<28 weeks gestation) and infants with extremely low birth weight (<1000 g) (Saigal and Doyle, 2008). However, preterm infants (PTIs; <37 weeks gestation) remain at high risk for development of disabilities, and recent research indicates they experience a broad and complex spectrum of adverse neurodevelopmental outcomes (Adams-Chapman et al., 2018). More specifically, PTIs have an increased risk of brain injury and disruption of brain maturation, which can manifest as an increased risk of cerebral palsy, cognitive deficits, and psychiatric disorders, such as attention deficit hyperactivity disorder or autism spectrum disorder. Moreover, PTIs may experience modifications of the hypothalamic-pituitary-adrenal (HPA) axis, although stimulation of the OXT system can protect against these modifications (Grunau et al., 2007). Several studies suggested that the impaired brain maturation of PTIs is at least partly a consequence of their atypical early-life environment, and their exposure to various stressors, such as physical pain and maternal separation (Anand and Scalzo, 2000; Grunau et al., 2006; Flacking et al., 2012).

This review examines the environmental risks factors to which PTIs are exposed during their early lives in NICUs, with a focus on exposure to pain and early maternal separation. We also review the impact of the long-term and simultaneous exposure to these risk factors on the OXT system. In everyday clinical practice, PTIs may undergo painful procedures while separated from their parents. It is possible that the simultaneous experience of these two negative early experiences – pain and parental separation – has a synergistic and negative impact on infant development. Similarly, it is possible that interventions which prevent these early negative experiences could have cumulative positive effects.

Thus, we also examine the effect of parental presence on protection against the short-term and long-term effects of pain in PTIs.

This review is needed as to date, no studies scrutinized the cumulative impact of separation and pain on the specific hormone of the OXT. Moreover, no reviews, at our knowledge, formulated a clear and evidence-based theoretical framework explaining the role of OXT in early family-based interventions.

The main reason for reviewing the role of OXT, instead of other hormones, is that OXT plays a pivotal role both in pain perception and early separation, which are both negative and stressful events that PTI in the NICU experience during the first weeks of hospitalization. OXT is involved in the attachment process and has analgesic properties, demonstrated in preclinical models, while other hormones (such cortisol) are specifically linked to stress or to the long-term effects of pain, but not specifically to attachment and early separation.

Our specific objectives were to examine the hypothesis that increased parental care using early vocal contact (EVC; Filippa et al., 2017a), and skin-to-skin-contact (SSC), provides benefits to PTIs undergoing painful procedures in the NICU, and that this effect is mediated by the OXT system. We also review the potential applications and opportunities for research in the field of risk aversion for neonatal pain and reduced parental care in PTIs, and propose protective actions that may help to improve the developmental outcomes of PTIs.

## Procedural Pain and Early Separation in the NICU

### Epidemiology of Procedural Pain and Early Separation in the NICU

Preterm infants (PTIs; <37 weeks gestation), especially very PTI (VPIs; 28–32 weeks gestation), and extremely preterm infant (<28 weeks gestation), are hospitalized in NICUs where they receive early postnatal exposure to an environment that differs markedly from the *in utero* environment (Kuhn et al., 2011). In particular, the NICU exposes them to excessive deleterious sensory stimuli, and deprives them of biologically meaningful sensory stimuli. The auditory environment of the NICU is characterized by frequent loud and high-pitched sounds, and this noise triggers stress responses, reduces physiological well-being, and disrupts sleep (Kuhn et al., 2012, 2013). The NICU also limits access to the vocal signature of the mother’s voice.

Furthermore, VPIs are frequently exposed to stressful and painful stimuli during care in the NICU. This burden results in a “distressing experience associated with actual or potential tissue damage with sensory, emotional, cognitive, and social components” (Williams and Craig, 2016), and still occurs in nearly all NICUs, although some recent epidemiological studies reported improvements in reducing procedural pain and improving analgesia (Simons et al., 2003; Carbajal et al., 2008, 2015; Johnston et al., 2011; Roofthooft et al., 2014; Allegaert and van den Anker, 2016). In France, the Epidemiology of Procedural Pain in Neonates study (EPIPPAIN 1), conducted in 2005, showed that each neonate experienced a mean of 10 painful procedures per day of hospitalization (Carbajal et al., 2008). A recent systematic review of observational studies that assessed painful procedures in neonates found that each newborn can undergo 7.5–17.3 procedures per day (Cruz et al., 2016). The most common procedures involve skin breakage and nasal or tracheal suctioning. Moreover, there are wide variations in the administration of analgesics to PTIs among countries, and among units in the same country (Carbajal et al., 2008). For example, a European prospective study (EUROPAIN) showed that the use of sedation or analgesia for PTIs varied from 0 to 100% among different centers (Carbajal et al., 2015). In addition, discomfort and stress related to human interventions during “standard” routine care, even supposedly non-painful procedures (diaper changes, temperature measurements, and mouth care), add to this burden (Sizun et al., 2002; Catelin, et al., 2005). Repeated exposure to odors that irritate the trigeminal system, such as those from health care products, can also trigger behavioral, and cortical pain responses (Frie et al., 2018). Routine nursing interventions can even potentiate the pain associated with many procedures. For example, blood collection taken immediately after routine clustered care provoked a greater pain response than blood collection following a period of rest (Holsti et al., 2006).

Very preterm infants also experience early and prolonged separation from their parents, and this separation is a well-known critical stressor for the infant (Flacking et al., 2012). Parental presence in a NICU is greater when there is implementation of infant- and family-centered developmental care, and when the NICU design supports increased parental presence by providing single family rooms (Lester et al., 2016). Studies that compared parental access and involvement of parents in the care of infants in Europe indicated increased parental presence in the Nordic countries (Greisen et al., 2009; Pallas-Alonso et al., 2012). Significant discrepancies remain among the NICUs in Europe, and there is evidence that providing parents the opportunity to stay overnight in a NICU prolongs the time they can spend with their newborns (Raiskila et al., 2017). Separation is also stressful to parents, and separated PTIs lack feedback from their parents, to whom they are normally sensorially tuned. Sensory interactions and biologically meaningful stimuli from the mother and father support early bonding and attachment (Flacking et al., 2012; Korja et al., 2012).

Repeated procedural pain and early parental separation by a PTI can also have negative consequences later in life.

### Long Term Consequences of Procedural Pain and Early Separation in the NICU

There is evidence that the NICU environment itself may interfere with the neurodevelopment and growth of VPIs. For instance, excessive noise contributes to the neurocognitive burden of PTIs (Graven, 2000; Wachman and Lahav, 2011), or excessive sensory deprivation and isolation in the incubator (Lagercrantz, 2016) may contribute to the neurocognitive burden of PTIs, and may lead to attention deficit hyperactivity disorder (Gray and Philbin, 2004) and/or alterations in early communication skills. In addition, the pain and stress that VPIs experience during care can impact brain growth and function (Smith et al., 2011). An increased exposure to invasive procedures during care of PTIs is associated with a lower IQ at school age (Vinall et al., 2014). Thus, untreated neonatal pain can impair multiple aspects of brain development, including cognition, emotional responses, and motor function. Receipt of analgesics is also associated with cognitive decline in these infants (Anand et al., 2004; Grunau et al., 2009), and excessive analgesia, such as during situations that are not painful, might increase these potential detrimental effects of analgesia. Thus, care strategies should attempt to reduce experiences of acute pain and unnecessary use of pharmacological analgesics. Another goal of pain management in neonates is to maximize the newborn’s capacity to cope with and recover from painful experiences (Carbajal et al., 2004).

Early separation of PTIs from their parents can also adversely impact their neurodevelopment, and can have adverse consequences later in life for the PTIs and their parents (Flacking et al., 2012). Parental separation limits the opportunities for early engagement in intimate contact with the parents (Bialoskurski et al., 1999). Early mother-infant separation also has a long-term impact on the infant’s autonomic, neuroendocrine, and immune systems (Sanchez et al., 2001). Over the long-term, this separation can alter the neurocognitive outcomes of PTIs, and their emotional experiences can negatively impact their emotional processing, and the health of the parents (Morelius et al., 2007; Korja et al., 2012; Montirosso et al., 2012a, 2014; Kommers et al., 2016). For instance, a recent study found that reduced bonding of mothers with VPI was associated with less intimacy with the infant, and with infant difficulties in regulation of socio-emotional stress at 3-months of age (Provenzi et al., 2017).

### Effects of Procedural Pain and Mother-Infant Separation on the OXT System

#### Procedural Pain and the OXT System

Pain associated with routine care or a medical procedure may alter the infant’s OXT system and have long-term effects (see section “Role of OXT in Modulation of Pain”). Although many studies have established the analgesic effects of OXT, little is known about the potential effect of chronic or procedural pain on regulation of the OXT system. One study showed that children with recurrent abdominal pain of psychosomatic origin had low concentrations of plasma OXT and high concentrations of cortisol (Alfvén et al., 1994). One approach to evaluate the association of the OXT system with pain is to analyze the impact of painful procedures on cortisol levels and the HPA axis, because these systems are known to interact with the OXT system. For instance, OXT can inhibit the function of the HPA axis at several levels during the production of cortisol (Neumann, 2002; Moberg and Prime, 2013): corticotrophin releasing factor release from the hypothalamus, release of adrenocorticotropic hormone from the anterior pituitary, and cortisol release into the circulation from the adrenal cortex.

Thus, additional studies of the HPA axis may provide a better understanding of the relationship of long-term exposure to pain with the OXT system. In animals, perinatal unpredictable stressors that stimulate the HPA axis also reduce OXT levels in the hypothalamus (Lee et al., 2007). Repeated exposure to pain is associated with increased activation of the HPA axis, and the OXT system can modulate this effect (Petersson et al., 1999). When OXT action is dampened in rodents by treatment with a selective OXT receptor antagonist, this reduces pair bonding and stimulates the HPA axis (Detillion et al., 2004). Thus, in animals, early and repeated exposure to pain has detrimental effects on behavior, presumably due to upregulation of the HPA axis. However, studies in humans indicated that a painful procedure in neonates could increase or have no impact on cortisol levels (Magnano et al., 1992; Herrington et al., 2004; Cignacco et al., 2009). A series of studies by Grunau et al. (2004, 2005) showed that exposure of PTIs to more neonatal pain had different effects on HPA responsiveness throughout development. In particular, cortisol responses were dampened while infants were still in the hospital with ongoing environmental stress, but then increased later during infancy.

Although there is strong evidence for the role of the OXT system in pain modulation, to our knowledge, no studies have yet evaluated the effect of early and repeated pain experience on the regulation of the OXT system in PTIs.

#### Early Mother-Infant Separation and the OXT System

Changes in the responsivity to social behaviors are critical determinants for development of bonding and attachment processes during the sensitive period soon after birth, and early separation of the infant from the mother has a negative impact on these processes (Hofer et al., 1993). However, the relationship of these changes with the OXT system is still uncertain. We examine this issue below by a review of animal and human studies that used different approaches to address this issue.

Numerous researchers have examined the impact of the OXT system on the early development of animals. For example, manipulating different crucial elements during early development indicated that the OXT system responded during periods of separation and reunion of mothers and offspring (Veenema, 2012). In rodents, long periods of early maternal separation led to reduced maternal care, and also affected regulation of the OXT system. At the physiological level, offspring that receive less maternal licking and grooming exhibit decreased estrogen-mediated up-regulation of OXT receptor binding and c-fos immunoreactivity in hypothalamic regions that are implicated in maternal care, such as the medial preoptic area (Champagne et al., 2001). This epigenetic regulation persists into adulthood and, in female offspring, can account for the intergenerational transmission of maternal behaviors (Maestripieri et al., 2007). Furthermore, the levels of OXT receptors in the central nucleus of the amygdala are significantly greater in females that receive more maternal care, regardless of their reproductive status (Francis et al., 2000).

Moreover, other studies of animal models (whose results have not yet been verified in humans) showed that rats who had only brief separations during early infancy had higher expression of OXT receptors than rats that received poor maternal care or more extended separations as pups (Lukas et al., 2010). Moreover, early maternal separation interferes with the healthy development of OXT receptors in specific forebrain regions, such as the agranular cortex (juveniles and adolescents), the lateral septum (adults), the caudate putamen (adults), but increased the level of OXT receptors in the ventromedial hypothalamus (adults) (Lukas et al., 2010). Maternal separation also increased or had little effect on OXT-immunoreactivity in the paraventricular nucleus of males (Tsuda et al., 2011), but led to a decreased OXT-immunoreactivity in the paraventricular nucleus of lactating and non-lactating adult females (Veenema et al., 2007).

Similarly, human studies showed that after a period of contact with infants, salivary OXT levels were only greater among mothers who had highly affectionate contact and among fathers who had highly stimulatory contact (Feldman et al., 2010). This led to the conclusion that higher levels of parental OXT are linked with higher levels of parental care. Thus, greater maternal contact soon after the birth of an infant leads to greater maternal production of OXT in experimental animals and humans (Francis et al., 2000; Feldman et al., 2010).

Administration of exogenous OXT after maternal separation or a stressful experience may provide protective effects and increase the resilience of mothers and infants (Barrett et al., 2015). More specifically, administration of OXT into the central amygdala increased the social competence of newborn rats after separation, and also reversed the effects of early prenatal stress (Lee et al., 2007). Another rat study demonstrated that maternal separation induced depressive-like behaviors in adult male mice, and that these behaviors were associated with abnormal mitochondrial function and immune-inflammatory responses in the hippocampus (Amini-Khoei et al., 2017). However, activation of the OXT system by OXT injection into the brain protected against the negative effects of maternal separation by altering the brain and behavior (Amini-Khoei et al., 2017).

Thus, these many studies suggest that regulation of the OXT system mediates the negative effects of an atypical early social environment (such as early maternal separation), and promotes pro-social behaviors. Additional human studies are necessary to improve our understanding of the impact of upregulation of the OXT system, after exogenous administration or endogenous production, on an infant’s brain and social behaviors the context of maternal contact.

### Role of OXT in Modulation of Pain

The role of OXT in pain modulation is now well-established, and it has putative action at almost every level of the pain pathway, including the peripheral, spinal, and supra-spinal systems. However, a recent review of clinical studies found that OXT reduced pain in adult subjects in only about half of all studies (Boll et al., 2018). Among the positive results, OXT reduced low back pain after intrathecal infusion (Yang, 1994), reduced visceral pain symptoms in patients with irritable bowel syndrome after intravenous injection (Louvel et al., 1996), and reduced headache in a dose-dependent manner after intranasal administration (Wang et al., 2013). Additional human studies of cortical integration in regions associated with the emotional dimension of pain expression using functional magnetic resonance imaging (fMRI) found that OXT modulated certain socio-emotional tasks (Herpertz and Bertsch, 2015; Wigton et al., 2015). These observations and others led to the recent proposal that OXT modulates several dimensions of pain expression, and had strong effects on emotional output, attentional processes, and social interactions (Tracy et al., 2015). However, there is still no direct and unambiguous link between pain expression and OXT. The few available imaging studies indicated that intranasal administration of OXT to humans reduced negative emotions related to pain from heat stimulation, and positively modulated responses related to empathy when viewing an emotional picture (Singer et al., 2008; Zunhammer et al., 2015, 2016). This result corresponds with the hemodynamic responses in emotion-processing brain structures, such as the amygdala (Zunhammer et al., 2015). In contrast to these human studies, there is unambiguous evidence that OXT has analgesic effects in many animal models of pain (Rash et al., 2014), and these studies have led to characterization of the underlying molecular and cellular mechanisms.

#### Mechanisms of Central Analgesia

Central nervous system analgesia first relies on axonal projections, which originate from the oxytocinergic hypothalamic neurons (in paraventricular, supraoptic, and accessory nuclei) and innervate many pain processing structures (Poisbeau et al., 2018). This includes the spinal cord, where OXT release onto second-order neurons selectively inhibits “pain messages” carried by nociceptive-specific C and Aδ-type sensory neurons in animal models (Rojas-Piloni et al., 2007, 2010; Eliava et al., 2016). In agreement, intrathecal or intracerebroventricular injections of OXT or a selective agonist of the OXT receptor substantially reduced the symptoms of pain in several animal models of inflammatory and neuropathic pain (Miranda-Cardenas et al., 2006; Russo et al., 2012; Eliava et al., 2016).

This likely results from an increased inhibition mediated by GABA_A_ receptors, and from an overall reduction in the excitability of spinal neurons that express the OXT receptor (Breton et al., 2008, 2009). Compared to the spinal cord, there are limited data regarding the effect of OXT on pain modulation in supraspinal structures although it is generally also associated with increases in GABAergic inhibition.

Interestingly, OXT modulation of GABA_A_ receptors is likely mediated by changes in intracellular phosphorylation (Brussaard and Koksma, 2003; Vergnano et al., 2007) and by establishment of an optimal chloride transmembrane gradient, because the action of OXT is mediated by chloride-permeable GABA_A_ receptor-channels. Recent studies indicated that OXT receptor signaling directly regulated the expression of the potassium-chloride transporter KCC2, which maintains low intracellular chloride concentrations in neurons, and ensures optimal GABA_A_ receptor-mediated inhibition (Tyzio et al., 2006; Leonzino et al., 2016). This discovery is of fundamental importance, because there is evidence that altered OXT levels and impaired chloride-mediated inhibitory control contribute to several neurodevelopmental disorders (Lefevre and Sirigu, 2016) and are responsible for the appearance and maintenance of neuropathic and inflammatory pain in adults (Price et al., 2009). Recent research indicated that separation of neonatal rats from their mothers leads to reduced OXT signaling, and this accounted for the development of nociceptive hypersensitivity and a failure of stress-induced analgesia during the postnatal development of pups and of mature rats (Melchior et al., 2018).

#### Mechanisms of Peripheral Analgesia

Several lines of evidence support the interpretation that OXT provides analgesia by acting on the peripheral nervous system, although some contradictory results indicate that vasopressin receptors might explain this effect (Schorscher-Petcu et al., 2010; Qiu et al., 2014). Juif and Poisbeau (2013) administered intravenous bolus injections of OXT and vasopressin to rats, and demonstrated that low physiological concentrations of both neurohormones reduced the number of action potentials carried by C-type nociceptors. However, simultaneous intravenous injection of an OXT receptor antagonist abolished these anti-nociceptive effects. This result is consistent with the results of optogenetic experiments which showed that selective stimulation of hypothalamic neurons increased the release of OXT into the bloodstream (Eliava et al., 2016). In agreement, release of OXT (but not vasopressin) into the blood of rats after an acute swim stress had an analgesic effect (Wotjak et al., 1998; Juif and Poisbeau, 2013). Despite some discrepancies, there is a broad consensus that OXT has analgesic effects on the peripheral nervous system. The target structures are unknown in most cases, although some recent results have suggested novel mechanisms.

Oxytocin can act on several different peripheral targets, including the skin. Skin cells express the OXT receptor and keratinocytes can produce and release OXT (Denda et al., 2012). In line with this observation, recent research showed that subcutaneous administration of OXT decreased neuronal firing of Aδ/C fibers (Gonzalez-Hernandez et al., 2017). This result could be explained by the expression of OXT receptors in non-peptidergic C-fiber cell bodies (Wrobel et al., 2011; Moreno-Lopez et al., 2013). The presence of OXT receptors in peripheral terminal axons of the skin (Gonzalez-Hernandez et al., 2017) is of particular interest, because touch-evoked OXT release by keratinocytes could explain the analgesia induced by stimulation of C tactile afferents (Walker et al., 2017) that occurs when newborns receive hand massages or perform sucking responses (Matthiesen et al., 2001). One possible mechanism could be membrane hyperpolarization of the sensory afferents, followed by increased intracellular calcium (Gong et al., 2015).

The most recent surprising results were from Nersesyan et al. (2017), who described the agonistic effect of OXT on TRPV1 channels, which are expressed by a subset of C nociceptors and are well-known for their role in responses to heat stimulation. These researchers identified the binding site for OXT on TRPV1 channels, and demonstrated that OXT blocks their function, thus explaining the analgesic effect of OXT.

To conclude, there is strong evidence that OXT has analgesic effects in the vast majority of animal models of pain (Rash et al., 2014). However, the evidence for this effect in humans needs further investigation, even if the recent over mentioned studies are encouraging.

Even though recent studies support the analgesic effects of OXT in humans, further studies of this topic are needed. Studies of PTIs indicated that OXT influences multiple psychological dimensions that impact the experience of pain, such as selective attention to pain, negatively valenced emotions, and social support. All of these are associated with neuronal activities in brain regions that are modulated by OXT administration and have roles in socio-emotional tasks.

## Role of OXT in Early Contact and Painful Procedures

### Effects of Parental Presence on Pain Management

Optimal pain management requires careful assessment and a combination of prevention and treatment by pharmacological and non-pharmacological methods. Parental interaction is an important non-pharmacological method for reducing pain in PTIs. Furthermore, some forms of interaction, such as SSC, breastfeeding, and EVC with familiar voices, must occur in an appropriate socio-emotional context and can be performed only by the mother and the father (except for breastfeeding). The efficacy of breast feeding and SSC are well established (Cignacco et al., 2007; Pillai Riddell et al., 2015). Thus, SSC alleviates pain responses to single painful procedures (such as a heel stick) (Mooncey et al., 1997; Johnston et al., 2017) and reduces cortical pain responses after venipuncture in PTIs (Olsson et al., 2016). The analgesic effect of SSC increases when it is given in combination with sweet solutions (Johnston et al., 2017). The optimal duration of SSC, the long-term impact of repeated SSC, and its interactions with other interventions require further investigation (Johnston et al., 2017). Breastfeeding is effective in diminishing mild procedural pain in neonates (Carbajal et al., 2003; Codipietro et al., 2008; Shah et al., 2012), and breast milk seems to be as effective as sweet solutions in relieving pain in full-term neonates (Watterberg et al., 2016). The odor of mother’s milk also appears to reduce pain from a heel stick in full-term neonates (Nishitani et al., 2009) and in PTIs (Baudesson de Chanville et al., 2017). Thus, parents, especially the mother, can help a neonate remain calm during painful procedures and to recover more rapidly by delivering appropriate sensory cues to the infant. Moreover, parental presence appears to be associated with reduced pre-procedural pain (Carbajal et al., 2008). In particular, the large EPIPPAIN 2 study reported that parental presence was associated with lower pain scores (DAN pain score <3) following venipuncture (Courtois et al., 2016a). The same study also reported that parental absence before a heel-stick was associated with a lack of pre-procedural analgesia (Courtois et al., 2016b). Although parental presence reduces procedural pain of infants, parents may need help in to developing coping strategies that reduce distress related to their infant’s pain (Franck et al., 2004, 2005).

Developmental care programs can help to reduce stress in the parents of PTIs. Infant- and family-centered developmental care programs aim to adapt the sensory environment of vulnerable newborns to their sensory abilities and expectations, and to integrate parents as the primary caregivers so they can better support infant well-being and neurodevelopment, and the bonding process. Infant pain management is an important component of infant- and family-centered developmental care, and has documented short- and long-term benefits (Montirosso et al., 2012b, 2016a,b). This holistic approach can support infant pain management through an architectural NICU design that supports parental presence, close observation of the infant, high involvement of parents as primary caregivers, and coordinated use of non-pharmacological methods for pain relief. Previous research has examined the impact of parental presence and NICU architectural design on pain management by comparing PTIs cared for in single family rooms or open-bay NICUs (Lester et al., 2014). PTIs in single family rooms received fewer medical procedures, received more parental care, and had less neonatal pain (based on Preterm Infant Pain Profile scores) as determined by the nursing staff of each shift. A precise and individualized evaluation of the signs of withdrawal and approach of each infant using Newborn Individual Developmental Care Program (NIDCAP) observations allow individualization of care procedures, with adjustment according to the tolerance of each child. Previous researchers have used NIDCAP cues to evaluate pain and have integrated them into different pain scores (Holsti et al., 2004; Holsti and Grunau, 2007; Lundqvist et al., 2014). The primary goals of infant- and family-centered developmental care are to reduce systematic and unnecessary procedures, and to support continuous parental involvement in the care and the evaluation of the infant. These programs also promote grasping opportunities and hand-to-mouth interactions to support the autonomy of the infant. These developmental care strategies can effectively reduce pain during and after routine care procedures (Sizun et al., 2002; Catelin et al., 2005). The newly proposed EValuation of INtervention scale allows evaluation of the use of non-pharmacological strategies to reduce pain and stress in the NICU (Warren et al., 2016). This scale helps caregivers to record different evidence-based best practices implemented before, during, and after routine care or painful interventions, and can potentially allow further evaluation of the impact of infant- and family-centered developmental care on infant pain management.

Individualized developmental care programs are also effective. For example, a prospective observational study in Netherlands indicated that implementation of NIDCAP-based stress reduction strategies was temporally associated with a significant decline in the mean number of painful interventions per NICU patient and per day (Roofthooft et al., 2014). Another study reported that the NIDCAP program decreased stress, pain-related behaviors, physiologic stress responses, and the use of sedatives and opioids (Westrup et al., 2007). A randomized clinical study reported that NIDCAP decreased stress responses due to painful procedures and the requirement for sedation (Heller et al., 1997). A randomized controlled trial of 36 PTI receiving 68 eye examinations reported that a NIDCAP-based intervention did not decrease pain responses, but led to faster recovery, as determined by lower salivary cortisol levels at 60 min after the examination (Kleberg et al., 2008).

Taken together, these studies support the benefits of parental presence, ideally *via* an infant- and family-centered developmental care program such as the NIDCAP, on infant pain management in the NICU.

### Maternal Contact and Regulation of Endogenous OXT

The most striking effect of OXT is its promotion of pro-social behaviors, and this is indirectly related to its neuroprotective effect during infant development. Two pioneering studies performed several decades ago (Pedersen et al., 1982; Fahrbach et al., 1984) first reported that maternal OXT is a crucial hormone for the regulation of prototypical social behaviors, such maternal behaviors.

A strong parent-infant bond supports the infant’s development and protects the infant from danger and stress. During the bonding process, there is an increase of typical maternal behaviors, such as affective and synchronized vocalizations, gazing, and touching. These early maternal behaviors shape the infant and are shaped by the infant. The first reciprocal interactions of the mother and infant have important effects on the infant’s brain structure and development, especially on the infant’s social, emotional, and cognitive competences, and can provide long-term protection against stress and pain (Meaney, 2001; Feldman and Eidelman, 2003, 2007).

Maternal behaviors in the bonding process are intuitive (Papoušek and Papoušek, 2002) and are tuned to the infant’s needs and requests. The bonding process is not a unilateral action, but is a reciprocal and bidirectional; infants are directed and shaped by maternal behaviors, and they actively engage mothers during their interactions. This mutual responsiveness between mothers and infants leads to mother-infant bonding in humans and animals (Nagasawa et al., 2012). Most mammalian infants produce a variety of cues to the mother, such as olfactory or auditory signals (Lévy et al., 2004; Ehret, 2005), that stimulate a range of maternal behaviors. For example, rodents mothers search for and retrieve their pups using vocalizations (Branchi et al., 2001).

Maternal separation induces changes in the reciprocal responsiveness of the mother and infant, and these are mediated by the OXT system. The intracerebral release of OXT, among other mechanisms, may mediate this response.

#### Association of OXT Level With Maternal Behaviors

The plasma OXT level of a pregnant woman is initially relatively stable, and then gradually increases as pregnancy progresses. The elevated plasma OXT level of the woman is associated with the expression of maternal behaviors soon after birth (Feldman et al., 2007; Levine et al., 2007). More specifically, an increase of the plasma OXT level between the first and the second trimester correlates with mother-infant bonding, and higher plasma and salivary levels of OXT occur in mothers who have more affectionate contact with their infants (Feldman et al., 2010). Similar to its role in other mammals, OXT supports bond formation in humans. In particular, OXT has roles in micro-level processes of parent-infant synchrony, in a parent’s attachments to his or her partner and infant, and in the parenting role and the parent-infant interaction.

Provision of appropriate maternal care increases OXT levels in the infant, and this affects brain organization early in life (Meaney, 2001). In particular, these behaviors increase OXT receptor binding in brain areas central to parenting, and the reward parents derive from their infants (Ross and Young, 2009). Caring behaviors are associated with OXT regulation, and there are increased levels of OXT in mothers and infants when the mothers provide comfort to their babies (Chisholm et al., 2005). Different types of maternal care (licking and grooming behaviors) are associated with increased levels of OXT receptors in brain regions previously known to regulate the expression of maternal behaviors in rats (Francis et al., 2002).

On the contrary, the intracerebroventricular infusion of a selective OXT antagonist into female rats disrupts the development of specific maternal behaviors, such as pup licking and adoption of the crouching posture used during nursing (Pedersen and Boccia, 2003). Rats reared a mother who expresses few maternal behaviors become anxious as adults (Caldji et al., 1998), and this is associated with hyperactivity of the HPA axis (Liu et al., 1997). Modulation of maternal behavior may also have a conditioning effect on the OXT system of progeny. These animal studies suggest that promotion of the OXT system may be an excellent strategy to prevent the impaired neurodevelopment from early and prolonged exposure to stress and pain.

### Parental Contact During Routine Painful Procedures Provides Protection by Stimulating the OXT System

Early social experiences can affect social behaviors during adulthood by modifying the OXT system (Meaney, 2001). In particular experiences of early contact or separation have long-term effects – even transgenerational effects – by modulating the OXT system.

In parallel, early and repeated painful experiences (especially in PTIs) induce long-term over-sensitization to pain and stress, and have significant consequences on infant social and emotional competencies. As with maternal separation, the OXT system also plays a crucial role in repairing and reconstructing the infant’s resilience in response to painful stimuli. Thus, clinical and maternal care can act by increasing the endogenous activation of the OXT system.

These results suggest that the care of PTIs should consider establishment of an appropriate ecological niche to promote infant development (Browne, 2017) and administration of individualized care (Als et al., 2004). A positive social environment, with experiences of the social interactions of daily life, continuously activates the OXT system. Interventions that sustain social engagement, especially when there is diminished mother-infant contact due to infant prematurity or postpartum depression (Feldman et al., 2010), can have a positive impact on the OXT systems of the infant and mother and on subsequent social and emotional competencies.

### Effect of Early Vocal Contact on Stress and Pain of Neonates

Recent research has shown that non-pharmacological analgesic interventions, such as SSC, can diminish the adverse outcomes associated with neonatal pain and reduced maternal care. In addition, Seltzer et al. (2010) demonstrated that infant-directed speech (“motherese”) led to increased peripheral OXT release in 6 year-old children who were exposed to a social stressor. Thus, vocalizations may be as important as skin-to-skin contact for the neuroendocrine regulation of social bonding in humans.

Recent animal studies have also identified the effect of social vocalizations on OXT regulation and social behaviors (Tops et al., 2011; Theofanopoulou et al., 2017). Interestingly, electrophysiological studies in mice reported activation of the mother’s auditory cortex in response to pup ultrasonic vocalizations (USVs), but no such activation in females not exposed to these USVs (Liu and Schreiner, 2007; Cohen et al., 2011). Two species of “singing mice” (*Scotinomys teguina* and *Scotinomys xerampelinus*), which have a complex vocal repertoire, exhibit high OXT receptor binding in brain regions related to social memory, including the hippocampus and medial amygdala (Campbell et al., 2009). Moreover, OXT null mutant mice were less vocal than wild-type controls during separations from the mother and peers (Winslow et al., 2000). Remarkably, OXT also mediates the response to acoustic social stimuli (Marlin et al., 2015). Furthermore, the injection of OXT into the hypothalamus increases the rate and duration of USVs by female hamsters, suggesting that OXT controls these USVs as a crucial component in the initiation or maintenance of social contact (Floody et al., 1998).

Given that a rat’s OXT receptors are very active in the auditory cortex of the mother, and are activated by USVs, it is plausible that reciprocal vocalizations or calls play a fundamental role in the mother-infant bonding, possibly by activating a dopaminergic response and activation of OXT receptors. Studies of 2 week-old *Octodon degus* rodents reported an increased density of the NMDA receptors in limbic brain areas at 3 days after 6 episodes of brief parental deprivation and exposure to an unfamiliar environment, and that parental vocalizations during the separation period suppressed this response (Ziabreva et al., 2003).

Moreover, behavioral observations indicated that parental vocalizations suppress the exploratory activity of rat pups, most likely through its “anxiolytic” effect (Braun et al., 2003). There is also evidence that parental vocal communications regulate the pup’s physical development (Poeggel and Braun, 1996; Braun and Scheich, 1997) and behavior, and presumably protect the pup from exposure to frightening situations and reduce the level of anxiety during stressful experiences, such separation or pain. During and after exposure to pain, maternal protective and consolatory vocal behaviors are essential for emotional recovery of offspring, even though these behaviors do not directly impact the origin of pain. OXT plays a crucial role in these consolatory behaviors (Burkett et al., 2016).

### Potential Effects of Early Vocal Contact on OXT Regulation

In light of these previous studies, early vocal contact (EVC) in the form of live maternal speech and songs, can be an effective method for reducing pain in infants who are undergoing medical procedures. EVC is an early family-based intervention with a high degree of contact, in which mothers and fathers speak and sing intimately with their preterm infant (Filippa et al., 2017b). This increases the PTI’s emotional and autonomic stability (Filippa et al., 2013) and reduces maternal anxiety (Arnon et al., 2014). The support from a music therapist can allow the PTI to engage in communicative musicality (Haslbeck, 2014) when they hear specific songs of kin (“lullabies”) (Loewy, 2015).

Moreover, EVC, as a form of live and dynamic musical contact, decreases an infant’s sensitivity to painful stimuli. Maternal singing is one of the most widespread forms of intuitive and nurturing music experiences among humans. This ubiquitous form of communication provides early social and communicative cues to the infant. It is finely tuned to the infant’s needs and expectancies. The infant is not merely a passive receiver, but experiences an active “call” for participation in a reciprocal musical play. Music can affect social interactions among humans, and Chanda and Levitin (2013) proposed that the OXT system plays a crucial role in this response. EVC is also an effective method because it is a social vocalization involving emotions.

#### OXT and Recognition of Vocal Emotions

It is well known that emotional prosody can affect socialization and the capacity of humans to infer the mental states of others, either implicitly, or explicitly (Grandjean et al., 2006). Many studies found that OXT plays a crucial role in improving recognition of emotions from vocalizations. For example, intranasal administration of OXT improves the recognition of emotions associated with different facial expressions (Domes et al., 2010; Shahrestani et al., 2013) and body postures (Bernaerts et al., 2016). Tops et al. (2011) suggested that individuals who have a specific OXT receptor polymorphism (GG genotype, rs53576), which presumably has stronger binding to OXT, have increased sensitivity to social processing and fewer difficulties in hearing and understanding people in the presence of background noise.

Similarly, Hovey et al. (2018) showed that activation of the OXT pathway, specifically the aryl hydrocarbon receptor nuclear translocator 2 (ARNT2) gene, is significantly associated with the ability to recognize audio-visual emotions. Other research showed that nasal administration of OXT specifically enhanced the ability to discern the emotional states of others, but not with inferring their beliefs. In particular, Aoki et al. (2014) performed a clinical double-blind, placebo-controlled, within-subject crossover trial of subjects with autism spectrum disorders, and found that intranasal OXT administration increased the rate of correctly inferring the social emotions of others, but not inferring their beliefs. Their imaging analysis also indicated that the right anterior insula, which was initially negatively correlated with difficulties in emotion inferences in these subjects, is significantly increased and correlated with the enhanced ability to infer the emotions of others following OXT administration. Furthermore, Hollander et al. (2007) reported improved recognition of emotion in vocalizations following OXT administration to patients with autism spectrum disorders. These findings thus establish relationships of vocal communication, social processing, and OXT level.

## OXT as a Neuroprotective Factor in the Development of Preterm Infants

In addition to the potential effect of EVC and OXT on reducing pain and stress in infants, there is also evidence that OXT acts as a direct neuroprotective factor during development of the infant brain, and that OXT has different mechanisms and potential molecular targets in this process.

### OXT and the GABA Switch in Early Life

The neonate’s brain is particularly vulnerable to excitotoxic damage, necessitating a balance between excitatory and inhibitory neurotransmission. OXT is responsible for the “developmental switch” in GABA polarity, in that it provides critical neuroprotective and analgesic effects that counteract postnatal excitotoxic damage (Ben-Ari, 2018). More specifically, GABA_A_ receptors are ligand-gated Cl^-^ channels, and the postsynaptic GABAergic signal regulates intracellular Cl^-^ concentration. OXT influences the intra-neuronal Cl^-^ level during the perinatal period by regulating the expression NKCC1 and KCC2 transporters. Down-regulation of NKCC1 and up-regulation of KCC2 initiates the postsynaptic GABAergic shift from depolarizing to hyperpolarizing just after birth.

Interestingly, the postnatal GABAergic shift is incomplete or delayed in several animal models of autism spectrum disorder (Deidda et al., 2015) and after gestational immune challenges that exacerbate symptoms of autism in animal models (Corradini et al., 2018). These findings led to the development of “neuro-archeology” by Y Ben-Ari during the last decade, which posits that the neurodevelopmental consequences of prematurity and the high vulnerability of the premature brain could be caused by the reduced or delayed GABA shift caused by an effect of OXT.

### OXT and Inflammation in Intrauterine Growth Restriction and Prematurity

The OXT neurons of the adult rat exist as a small population of about 30 parvocellular neurons in the paraventricular nucleus of the hypothalamus, and these coordinate the peripheral and spinal release of OXT, and limit the symptoms of inflammatory pain (Eliava et al., 2016).

The OXT-mediated prevention of inflammatory pain may be extended to the entire central nervous system, and may be particularly relevant to intrauterine growth restriction and premature birth. Indeed, inflammation in the central nervous system plays a crucial role in the pathophysiology of perinatal brain damage in animal models and human neonates (Hagberg et al., 2012). Abnormal microglial activation induces white matter damage, neurocognitive disabilities, and neuropsychiatric disorders in children and adults (Rezaie and Dean, 2002; Leviton et al., 2005). A recent study used a double-hit rat model of perinatal brain injury induced by a low protein gestational diet and potentiated by postnatal injections of sub-threshold doses of IL1β (Mairesse et al., 2018). The results showed that systemic postnatal administration of carbetocin (a selective, long lasting, and brain diffusible OXT receptor agonist) reduced microglial activation at the transcriptional and cellulat levels, and provided long-lasting neuroprotection. Carbetocin treatment also had beneficial effects on myelination, long-term intrinsic brain connectivity, and behavior. Thus, targeting OXT signaling in the developing brain may be an effective approach to prevent neuroinflammation-induced brain damage that originates during the perinatal period.

## Conclusion and Perspectives

Oxytocin is a neuropeptide hormone that functions in the physiological responses to pain and stress (Neumann et al., 2000) and promotes prosocial behaviors (Carter, 1998). In particular, during the early period after birth, OXT regulates maternal behaviors (Pedersen, 1997) by promotion of social interactions and positive emotions (Uvnas-Moberg, 1998). Inhibition of OXT receptors or a decrease in OXT production, such as following separation of the mother and infant or stress during the critical neonatal period, correlate with poor maternal behaviors, and this has long-term negative consequences on the prosocial behaviors of mothers and infants. Animal studies have documented the protective effect of OXT administration, in that a single dose can reverse the effects of maternal separation and the many adverse sequelae in rodent pups (Lee et al., 2007). PTI often experience early maternal separation and painful events or procedures in the NICU, and these two risk factors are often simultaneous and appear to interact synergistically.

Positive social interactions can suppress internal physiologic systems that are activated by stress, and stimulate other internal systems that attenuate stress. OXT plays a crucial role in the attenuation of stress by enhancing the buffering effect of social support on stress responsiveness (Heinrichs et al., 2003). The cumulative effects of early maternal contact and an increased level of OXT can protect PTIs against many sequelae of early maternal separation and their painful experiences during their first weeks of life in the NICU. In this context, the maternal voice can have positive effect on infant recovery from stressful events (Seltzer et al., 2010).

**FIGURE 1:**
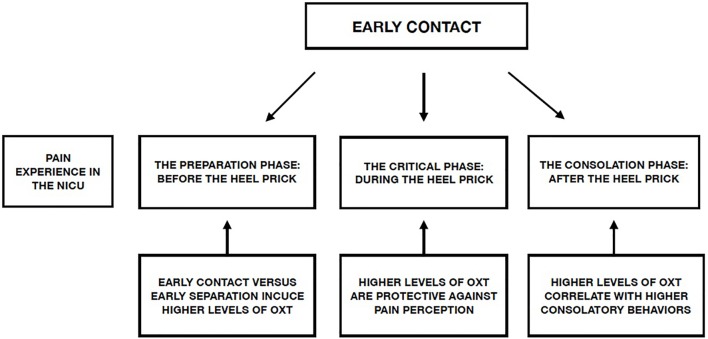
Possible effect of early contact on the different phases of a preterm infant’s experience of pain in the NICU and role of the OXT system.

Creating an environment that decreases the negative effects associated with preterm birth is one of the main aims of individualized developmental care in the NICU. Implementation of a series of protective actions during the different stages of painful procedures, mediated by the OXT system, can reduce the impact of these procedures on PTIs during their time in the NICU (Figure 1).

In light of the many studies reviewed here, we suggest the following protective actions for pain management in the NICU:

- Active involvement of parents with the infant during all phases of painful procedures in the NICU, including the
- preparation phase, the phase of acute pain, and the consolatory/reunion phase;
- Active involvement of nursing staff in supporting parental involvement with their infants during the preparation and consolatory phases of painful NICU procedures;
- Use of EVC as a non-pharmacological intervention to encourage contact between parents and PTIs, by use of live and directed speech and songs directed to the PTIs.

A limitation of this review is the lack of human studies, especially on the impact of pain and early maternal separation on the OXT system. Moreover, additional human studies are necessary to improve our understanding of the impact of exogenous administration – or endogenous production – of OXT on an infant’s brain and social behaviors.

Finally, further research is needed to investigate the impact of early contact between parents and infants in the NICU on regulation of the OXT system. Identification of the role of the OXT system during stressful conditions, such as painful procedures in the NICU, seems to be a particularly promising topic for future research.

## Author Contributions

MF and PK contributed to conception and design of the review. MF wrote the first draft of the manuscript. PP, JM, PH, MM, OB, and PK critically revised the manuscript for important intellectual content. PP, JM, DG, and PK wrote sections of the manuscript. All authors contributed to manuscript revision, read, and approved the submitted version.

## Conflict of Interest Statement

The authors declare that the research was conducted in the absence of any commercial or financial relationships that could be construed as a potential conflict of interest.

## Abbreviations

OXT: oxytocin
PTI: preterm infants
VPI: very preterm infants
